# GABA receptor modulation of arcuate kisspeptin neuron bursting and synchronization activity in female mice

**DOI:** 10.1111/jne.70170

**Published:** 2026-03-30

**Authors:** Toby Eddleston, Paul G. Morris, Allan E. Herbison

**Affiliations:** ^1^ Department of Physiology, Development and Neuroscience University of Cambridge Cambridge UK

**Keywords:** arcuate nucleus, GABA, kisspeptin, luteinizing hormone, synchronization

## Abstract

The arcuate nucleus kisspeptin (ARN^KISS^) neurons intermittently synchronize their activity to operate as the GnRH pulse generator and drive pulsatile reproductive hormone secretion in mammals. Although ARN^KISS^ neurons are known to receive various GABAergic inputs, the effects of GABA_A_ and GABA_B_ receptor modulation on their ability to synchronize remain unknown. We have used GCaMP6s to monitor the activity of multiple ARN^KISS^ neurons simultaneously in acute brain slices from diestrous female Kiss1‐Cre1,Ai162D mice. The effects of modulating GABA_A_ and GABA_B_ receptors on calcium transients exhibited by individual ARN^KISS^ neurons, reflecting burst firing, and their ability to generate synchronous bursting events were examined. The application of GABA was found to robustly suppress the occurrence of individual calcium transients and population bursting. The GABA_A_ receptor agonist muscimol had a biphasic effect in which ARN^KISS^ neurons could initially respond with an increase in baseline calcium but then became inhibited with significantly reduced episodes of individual burst firing and population synchronization events. Baclofen, the GABA_B_ receptor agonist, also reduced the frequency of ARN^KISS^ neuron burst firing. The receptor antagonists bicuculline and CGP‐35348 had no effects on individual bursting frequency or dynamics, or the synchronous activation of ARN^KISS^ neurons indicating a lack of ongoing GABA transmission in the acute brain slice. These observations show that while GABAergic activation may initially facilitate excitability through GABA_A_ receptor‐mediated depolarization, sustained GABAergic input to ARN^KISS^ neurons suppresses their burst firing and ability to synchronize through both GABA_A_ and GABA_B_ receptors. As such, GABAergic inputs to ARN^KISS^ neurons appear to have considerable potential to modulate the frequency of pulse generator activity in female mice.

## 1 INTRODUCTION

The arcuate nucleus kisspeptin (ARN^KISS^) neurons operate as the gonadotropin‐releasing hormone (GnRH) pulse generator and drive the pulsatile pattern of luteinizing hormone (LH) secretion required for mammalian fertility.^1^, ^2^ The great majority of ARN^KISS^ neurons synchronize their activity for periods of approximately 90 s every few minutes or hours to evoke pulsatile GnRH release into the pituitary portal system.^3^, ^4^ The precise mechanisms through which ARN^KISS^ neurons synchronize their activity remain under investigation as do the ways in which afferent inputs are able to control the intervals between these synchronization events (SEs).^1^ It is currently thought that emergent glutamate‐AMPA receptor signaling between reciprocally connected ARN^KISS^ neurons is the key driver of their synchronicity with the co‐expressed neuropeptides neurokinin B and dynorphin operating as neuromodulators.^3^, ^5^ Afferent inputs such as those provided by noradrenaline are able to influence SE frequency by directly hyperpolarizing ARN^KISS^ neurons and making it more difficult for the network to achieve emergent excitation.^6^

There are likely to be multiple afferent inputs to ARN^KISS^ neurons that could utilize GABA to modulate SE frequency.^7^, ^8^, ^9^ However, the role of GABA in modulating ARN^KISS^ neuron synchronization behavior has not been examined to date. Importantly, brain slice electrophysiological studies have demonstrated that individual ARN^KISS^ neurons exhibit GABA_A_ receptor‐mediated postsynaptic currents (PSCs) and that their dynamics can change across puberty, time of day, and depend on prior estradiol concentrations.^10^, ^11^, ^12^ The electrophysiological studies of DeFazio and colleagues revealed that GABA_A_ receptor activation had a depolarizing effect on ARN^KISS^ neuron membrane potential, although it was suggested that this was likely to be in the sub‐threshold range and may not result in the direct activation of firing.^11^ In contrast, Gottsch and colleagues found that both GABA and muscimol inhibited ARN^KISS^ neuron firing in brain slices from ovariectomized mice.^13^ Whether GABA_A_ receptor modulation may impact the synchronicity of ARN^KISS^ neurons remains unknown and the influence of GABA_B_ receptor signaling on individual ARN^KISS^ neurons or their synchronization behavior has not been investigated.

Using GCaMP imaging in an acute brain slice preparation we are able to monitor the burst firing behavior of multiple individual ARN^KISS^ neurons simultaneously and also observe their ability to generate intermittent synchronizations termed “miniature synchronization events” (mSEs). These mSEs are detected when recording from ~15 to 25 ARN^KISS^ neurons in the acute brain slice and are thought to be the building blocks of the large‐scale synchronization events occurring in vivo that drive pulsatile luteinizing hormone (LH) secretion.^3^ In the present study we questioned the impact of GABA_A_ and GABA_B_ receptor activation and inhibition on ARN^KISS^ neuron bursting and mSE generation in brain slices prepared from intact diestrous female mice.

## 2 METHODS

### 2.1 Animals

Mice were generated and housed as detailed previously.^3^ This involved crossing the 129S6Sv/Ev C57BL/6 Kiss1^Cre/+^ and C57BL/6 Ai162 (TIT2L‐GC6s‐ICL‐tTA2)‐D Cre‐dependent GCaMP6s lines (JAX stock #031562)^14^ with mice group‐housed in cages with environmental enrichment under conditions of controlled temperature (22 ± 2°C) and lighting (12 h light/12 h dark cycle; lights on at 7:00 h and off at 19:00 h) with ad libitum access to low phytoestrogen food (Teklad 2016, Envigo RMS, UK) and water. All animal experimental protocols were approved by the University of Cambridge, UK (P174441DE). Regularly cycling adult female mice (10–24 weeks old) were used for experiments on diestrus.

### 2.2 Brain slice preparation

Brain slices from the mid‐caudal ARN were prepared exactly as reported previously.^3^ Kiss1‐Cre,Ai162D mice were anaesthetized using isoflurane, decapitated, and the brain removed into oxygenated, ice‐cold slicing solution composed of (mM): NaCl 52.5; sucrose 100; glucose 25; NaHCO_3_ 25; KCl 2.5; CaCl_2_ 1; MgCl_2_ 5; NaH_2_PO_4_ 1.25; kynurenic acid 0.1 (95% O_2_/5% CO_2_). Coronal slices containing the ARN were prepared at 320 μm thickness using a VT1200S tissue slicer (Leica Biosystems UK) before being transferred to a submersion chamber containing an oxygenated (95% O_2_/5% CO_2_) aCSF recording solution composed of (mM): NaCl 124; glucose 30; NaHCO_3_ 25; KCl 3.5; CaCl_2_ 1.5; MgCl_2_ 1; NaH_2_PO_4_ 0.5 and held at 30°C for 1–5 h prior to use.

### 2.3 Brain slice calcium imaging and analysis

Brain slices containing the middle and caudal levels of the ARN were transferred to the stage of an Olympus BX51WI upright microscope with differential interference contrast optics, and constantly perfused with oxygenated aCSF at 30 ± 1°C. The intracellular calcium concentration of ARN^KISS^ neurons was estimated by recording their GCaMP6s fluorescence using a Prime BSI Express sCMOS camera (Teledyne Photometrics UK) and CoolLED pE‐300 ultralight source via an Olympus 40× immersion objective and GFP filter cube (Chroma). The excitation waveband was 470–490 nm, applied at 2 Hz for 100 ms, and emission collected at 500–520 nm with a 495 nm long‐pass filter. For analysis, ImageJ (v1.53c) was used to obtain mean fluorescence intensities over the image time series: active cell somata were selected manually as regions of interest (ROIs), and for each, the mean fluorescence values of a nearby background ROI were subtracted. Fluorescence intensity data and all metrics described below were analyzed using custom Python scripts. The change in fluorescence (ΔF/F) was calculated and individual calcium events in cells and population mSEs were registered as previously described.^3^ Briefly, events are recorded where the ΔF/F trace exceeded 2 standard deviations (SDs) above the trace mean. An mSE exists when the peak of calcium events from at least two neurons occurs within 10 s of each other. Event and mSE rates are presented as “per cell, per hour,” thereby controlling for the variation in the number of neurons being recorded in each brain slice.

For each experiment, a pre‐drug baseline was obtained, followed by a drug application period and a wash period. All of these measurement periods, from which event and mSE rates were calculated, were 12 min in duration and followed/preceded by a two‐minute gap to allow wash‐in/out of compounds. The mean number of neurons taking part in mSEs is reported for each measurement period, with this metric requiring at least one mSE to report a value; therefore, n is occasionally lower than that reported for event/mSE rates for the same experimental group.

The dynamics of individual calcium events were obtained as described previously^3^: briefly, a trace baseline value was calculated as the median value of all data points below a threshold of 0.5 SD above the trace mean. Each datapoint shown is a mean of means, representing one slice/experiment. A minimum of three neurons with paired calcium events in both the pre‐drug and drug‐applied periods was required to form a datapoint for each experiment. Half width is defined as the width of the calcium event at 50% amplitude from the calculated baseline to the peak. Rise time is the length of time taken to rise from 20% to 80% of maximum event amplitude, and decay time is the reverse.

To illustrate consistent phenomena in the traces within experimental groups, background‐subtracted fluorescence data were taken for every neuron across all slices in that group and a population mean trace created. Any remaining effects of very slow drift or photobleaching were accounted for by nonlinear least squares fit of an exponential‐linear baseline and subtracting this from the trace.

### 2.4 Compounds

Muscimol and baclofen were obtained from Fisher Scientific UK. Bicuculline was obtained from Merck UK, CGP‐35348 from Abcam UK and GABA from Bio‐Techne UK. Standard laboratory salts were purchased from Fisher Scientific UK.

### 2.5 Quantification and statistical analyses

Statistical analyses were performed in Prism 10 (GraphPad software Inc.). All values given in the text and within figures are mean ± SEM, and significance is defined as *p* < .05*, *p* < .01**, *p* < .001***, or *p* < .0001****. Paired statistical tests were employed to compare only the pre‐drug and drug‐applied metrics within the same experiment. The Shapiro–Wilk normality test was used to assess the distribution of datasets; however, no datasets met all criteria for parametric analysis, and so the Wilcoxon signed‐rank test was used to analyze all data. All analyses were two‐tailed, and all experiments replicated in a minimum of five animals per experimental group. Within each group, n slices = n animals.

## 3 RESULTS

### 3.1 Spontaneous GCaMP calcium events and synchronizations

We have previously reported that 98% of ARN^KISS^ neurons express GCaMP6 and that ~80% of GCaMP cells are immunoreactive for kisspeptin in this Kiss1‐Cre,Ai162D mouse line.^3^ Mid to caudal ARN coronal brain slices were prepared from diestrous‐stage female Kiss1‐Cre,Ai162D mice, with 8–29 kisspeptin neurons visible within the focal plane for simultaneous GCaMP fluorescence imaging. In the pre‐drug period (38 slices from 23 mice), individual calcium events, representing episodes of ARN^KISS^ neuron burst firing,^3^ occurred at a rate of 13.4 ± 1.4 events/cell/h, with mSEs occurring at 2.6 ± 0.3 mSEs/cell/h, and with 3.7 ± 0.2 cells (29.1%) contributing to each mSE. No relationship was found between the age of the diestrus‐stage mice and mean mSE rate (Spearman *r* = −0.09; *p* = .60; *n* = 38).

### 3.2 Effects of GABA on ARN^KISS^ neuron transients and mSEs

**FIGURE 1:**
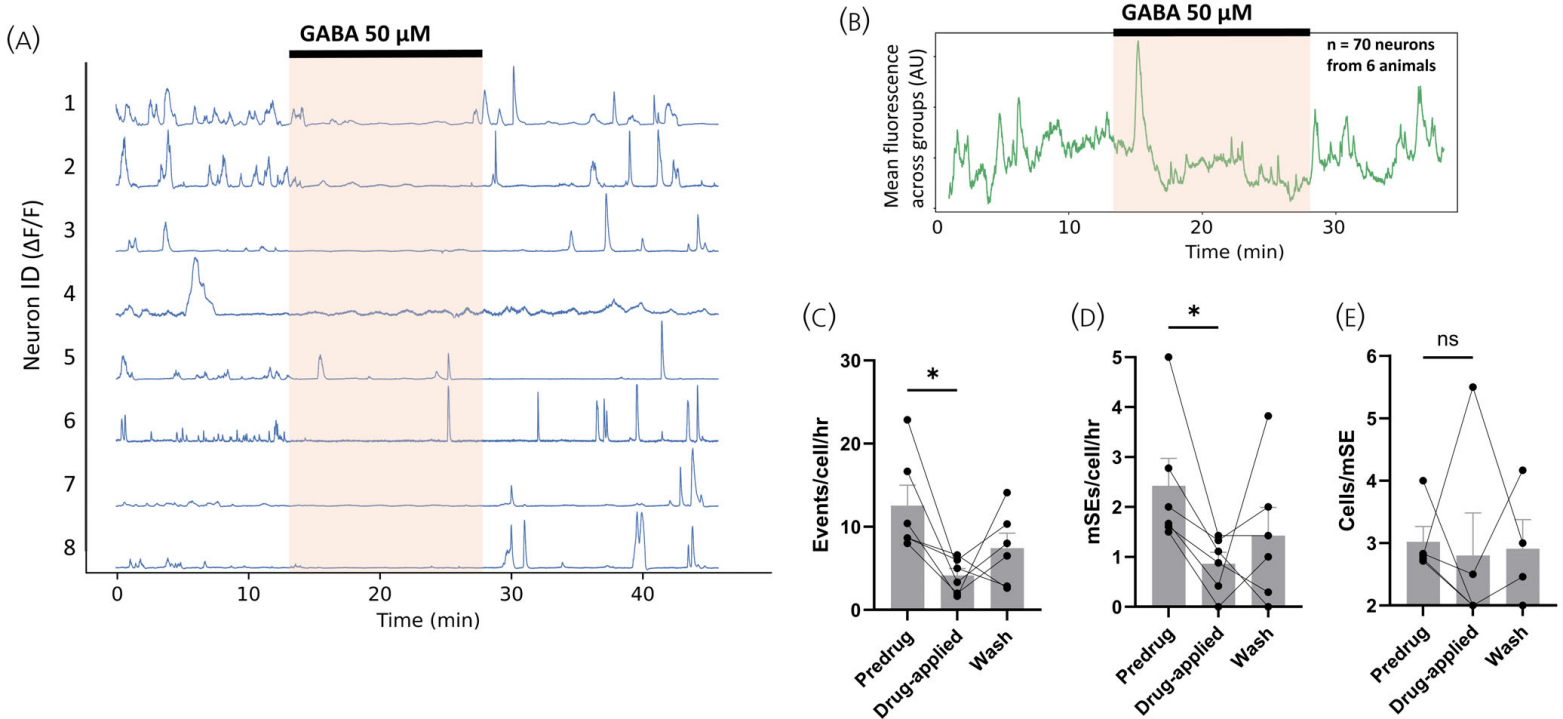
GABA robustly suppresses the spontaneous bursting of ARN^KISS^ neurons. (A) Representative ΔF/F GCaMP6s fluorescence recorded simultaneously from 8 ARN^KISS^ neurons. Following a baseline period, GABA (50 μM) was applied for 14 min (orange shading) followed by a wash period. (B) Baseline fit‐subtracted population trace showing the mean calcium activity of every neuron across all slices in the experimental group. (C–E) Histograms showing the effect of GABA on (C) mean + SEM number of calcium events/cell/h (*p = .0312, Wilcoxon, *n* = 6 slices from 6 animals), (D) mean + SEM number of mSEs/cell/h (*p = .0312, Wilcoxon, *n* = 6 slices from 6 animals) and (E) mean + SEM number of neurons contributing to synchronization events (*p* = .312, Wilcoxon; *n* = 5 slices from 5 animals). Each datapoint represents mean population activity within a single slice/mouse. mSE = miniature synchronization event.

In six slices (6 mice), application of GABA (50 μM) resulted in a pronounced reduction in the frequency of individual calcium events (12.6 ± 2.4 events/cell/h pre‐drug, 4.1 ± 0.9 events/cell/h in GABA; *p* = .0312; Wilcoxon, *W* = −21) as well as a reduction in the occurrence of mSEs (2.4 ± 0.5 mSEs/cell/h pre‐drug, 0.9 ± 0.2 mSEs/cell/h in GABA; *p* = .0312; Wilcoxon, *W* = −21) (Figure 1A–D). One experiment had a very large synchronization at the start of the drug‐applied period, which is visible in the overall group mean fluorescence trace (Figure 1B). The mean numbers of ARN^KISS^ neurons contributing to each mSE were unchanged in the presence of GABA (28.3%, 3.0 ± 0.2 cells/mSE pre‐drug and 23.3%, 2.8 ± 0.7 cells/mSE in GABA; *n* = 5; *p* = .625; Wilcoxon, *W* = −5) (Figure 1E).

### 3.3 Effects of GABA_A_ and GABA_B_ receptor agonists on ARN^KISS^ neuron transients and mSEs

**FIGURE 2:**
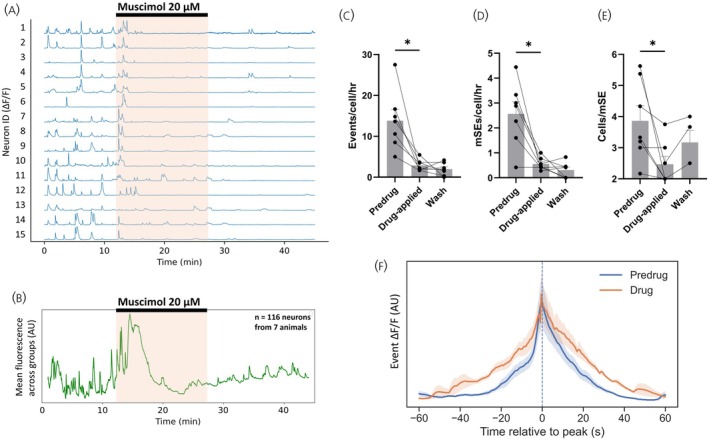
GABA_A_ receptor activation suppresses the spontaneous bursting and synchronization activity of ARN^KISS^ neurons. (A) Representative ΔF/F GCaMP6s fluorescence recorded simultaneously from 15 ARN^KISS^ neurons in a brain slice preparation that maintains spontaneous synchronized activity within the ARN^KISS^ neuron network (mSE, miniature' synchronization event). Following a baseline period, the GABA_A_ receptor agonist muscimol (20 μM) was applied for 14 min (orange shading) followed by a wash period. (B) Baseline fit‐subtracted population trace, otherwise unprocessed, showing the mean calcium activity of every neuron across all slices in the experimental group. (C–E) Histograms showing the effect of muscimol (20 μM) on (C) mean + SEM number of calcium events/cell/h (*p = .0156, Wilcoxon; *n* = 7 slices from 7 animals), (D) mean + SEM number of mSEs/cell/h (*p = .0312, Wilcoxon; *n* = 7 slices from 7 animals) and (E) mean + SEM number of neurons contributing to mSEs (*p = 0.0312, Wilcoxon; *n* = 7 slices from 7 animals). Each datapoint represents mean population activity within a single slice/mouse. (F) Mean profile of all GCaMP calcium transients recorded pre‐drug and during muscimol. Only those slices exhibiting transients in both conditions were used (*N* = 5).

Application of muscimol (20 μM) to seven slices (7 mice) for 14 min resulted in an overall pronounced reduction in the frequency of individual calcium events (13.8 ± 2.7 events/cell/h pre‐drug, 2.8 ± 0.5 events/cell/h in muscimol; *p* = .0156; Wilcoxon, *W* = −28) as well as a reduction in the occurrence of mSEs (2.6 ± 0.5 mSEs/cell/h pre‐drug, 0.6 ± 0.1 mSEs/cell/h in muscimol; *p* = .0312; Wilcoxon, *W* = −21) (Figure 2A–D). The number of ARN^KISS^ neurons contributing to each mSE was also significantly reduced from 30.5% to 17.6% (3.9 ± 0.5 cells/mSE pre‐drug, 2.2 ± 0.4 cells/mSE in muscimol; *p* = .0312; Wilcoxon, *W* = −21) (Figure 2E). Although a strong overall suppression of ARN^KISS^ neuronal activity was observed, it was evident that the entry of muscimol into the bathing solution resulted in an immediate increase in the baseline GCaMP signal in some ARN^KISS^ neurons before they became less active (Figure 2A). To visualize this effect as a group, we determined the overall mean detrended GCaMP fluorescence levels from all recorded neurons (*n* = 116) over all seven animals. This highlighted a robust increase in baseline GCaMP fluorescence upon entry of muscimol to the bath that persisted for several minutes before levels fell below baseline and then recovered upon washout (Figure 2B). The mean waveform of individual events in the presence of muscimol was altered leading to a broader profile (Figure 2F). This change in event shape metrics is quantified in Table 1, with half‐width and decay time significantly increased.

**TABLE 1:** Dynamics of individual calcium events (mean ± SEM) in the absence and presence of the various GABA receptor compounds.

	Half‐width (s)	Rise time (s)	Decay time (s)	Number of slices
GABA				*n* = 6
Pre‐drug	27.85 ± 3.45	10.73 ± 1.79	12.27 ± 1.64	
Drug	31.08 ± 4.35	8.63 ± 2.53	14.69 ± 2.96	
*p*‐value	.2188	.8438	.6875	
Muscimol				*n* = 6
Pre‐drug	25.9 ± 4.00	7.98 ± 1.49	11.20 ± 1.43	
Drug	40.36 ± 6.97	12.15 ± 3.16	15.50 ± 1.68	
*p*‐value	.0312	.3125	.0312	
Baclofen				*n* = 5
Pre‐drug	27.40 ± 3.61	8.43 ± 1.74	13.24 ± 1.98	
Drug	31.50 ± 5.48	8.13 ± 2.22	12.78 ± 3.10	
*p*‐value	.6250	.8125	>.9999	
Bicuculline				*n* = 6
Pre‐drug	22.81 ± 4.92	6.92 ± 1.58	11.35 ± 1.51	
Drug	26.08 ± 5.08	6.46 ± 1.68	13.33 ± 1.34	
*p*‐value	.8438	.8438	.5625	
CGP‐35348				*n* = 5
Pre‐drug	27.90 ± 10.34	10.26 ± 4.34	12.62 ± 3.92	
Drug	27.18 ± 3.05	6.19 ± 0.95	11.34 ± 1.56	
*p*‐value	>.9999	.4375	.6250	

*Note*: Number of slices equals number of animals.

**FIGURE 3:**
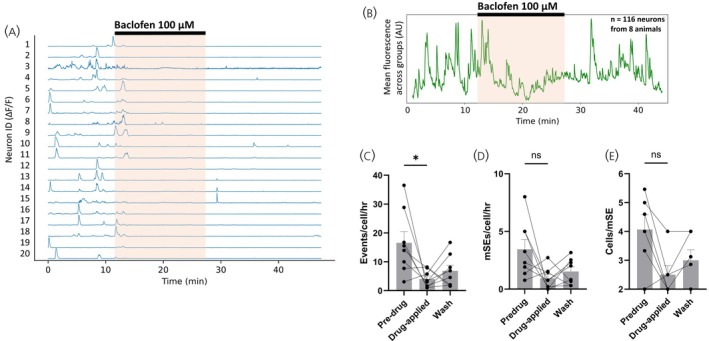
GABA_B_ receptor activation suppresses the spontaneous bursting of ARN^KISS^ neurons. (A) Representative ΔF/F GCaMP6s fluorescence recorded simultaneously from 20 ARN^KISS^ neurons. Following a baseline period, the GABA_B_ receptor agonist baclofen (100 μM) was applied for 14 min (orange shading) followed by a wash period. (B) Baseline fit‐subtracted population trace showing the mean calcium activity of every neuron across all slices in the experimental group. (C–E) Histograms showing the effect of baclofen (100 μM) on (C) mean + SEM number of calcium events/cell/h (*p = .0391, Wilcoxon; *n* = 8 slices from 8 animals), (D) mean + SEM number of mSEs/cell/h (*p* = .0781, Wilcoxon; *n* = 8 slices from 8 animals) and (E) mean + SEM number of neurons contributing to synchronization events (*p* = .0625, Wilcoxon; *n* = 6 slices from 6 animals). Each datapoint represents mean population activity within a single slice/mouse. mSE, miniature synchronization event.

The application of baclofen (100 μM) to eight slices (8 mice) for 14 min resulted in an overall reduction in the frequency of calcium events exhibited by individual ARN^KISS^ neurons (16.6 ± 3.9 events/cell/h pre‐drug, 4.0 ± 1.0 events/cell/h in baclofen; *p* = .0391; Wilcoxon, *W* = −30) (Figure 3A,C). However, only a nonsignificant trend was found for a decrease in mSEs rate (3.5 ± 0.9 mSEs/cell/h pre‐drug, 0.9 ± 0.3 mSEs/cell/h in baclofen; *p* = .0781; Wilcoxon, *W* = −26) with 6 of 8 slices exhibiting a reduction and two a small increase (Figure 3D). Similarly, the number of ARN^KISS^ neurons contributing to each mSE was reduced from 28.5% to 16.3% but did not reach significance (4.1 ± 0.5 cells/mSE pre‐drug, 2.5 ± 0.3 cells/mSE in baclofen; *p* = .0625; Wilcoxon, *W* = −19) (Figure 3E). In the case of baclofen, no consistent increase in baseline GCaMP signal was detected upon entry to the bath (Figure 3B).

### 3.4 Effects of GABA_A_ and GABA_B_ receptor antagonists on ARN^KISS^ neuron transients and mSEs

**FIGURE 4:**
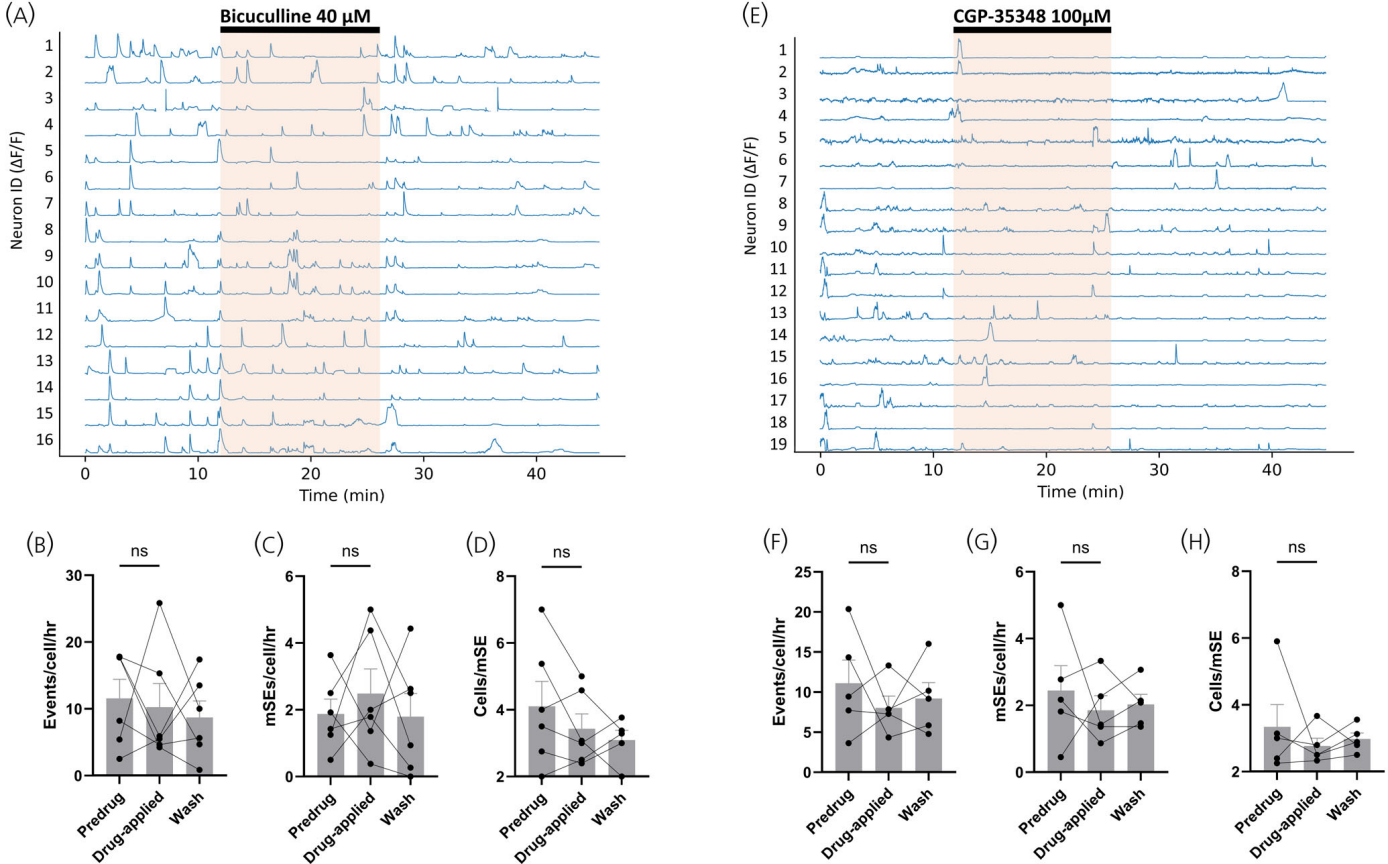
Endogenous GABA transmission does not modulate spontaneous ARN^KISS^ activity in the brain slice. (A) Representative ΔF/F GCaMP6s fluorescence recorded simultaneously from 16 ARN^KISS^ neurons in an acute brain slice. Following a baseline period, the GABA_A_ receptor antagonist bicuculline (40 μM) was applied for 14 min (orange shading) followed by a wash period. (B–D) Histograms showing the effect of bicuculline (40 μM) on (B) mean + SEM number of calcium events/cell/h (*p* = .6875, Wilcoxon; *n* = 6 slices from 6 animals), (C) mean + SEM number of mSEs/cell/h (*p* = .6875, Wilcoxon; *n* = 6 slices from 6 animals), and (D) mean + SEM number of neurons contributing to synchronization events (*p* = .4688, Wilcoxon; *n* = 6 slices from 6 animals). (E) Representative ΔF/F GCaMP6s fluorescence recorded from 11 ARN^KISS^ neurons with a 14 min application of GABA_B_ receptor antagonist CGP‐35348 (100 μM; orange shading). (F–H) Histograms showing the effect of CGP‐35348 (100 μM) on (F) mean + SEM number of calcium events/cell/h (*p* = .6250, Wilcoxon; *n* = 5 slices from 5 animals), (G) mean + SEM number of mSEs/cell/h (*p* = .8125, Wilcoxon; *n* = 5 slices from 5 animals), and (H) mean + SEM number of neurons contributing to mSEs (*p* = .6250, Wilcoxon; *n* = 5 slices from 5 animals). mSE, miniature synchronization event.

As some GABAergic afferents to ARN^KISS^ neurons are likely to arise from within the ARN, and significant spontaneous GABA_A_ receptor activity is recorded from ARN^KISS^ neurons in coronal brain slices,^10^, ^11^ we examined the impact of ongoing GABAergic transmission with receptor antagonists. In six slices (6 mice), the application of bicuculline (40 μM) resulted in variable effects on ARN^KISS^ neurons with no overall significant change (Figure 4A). The numbers of calcium events exhibited by individual ARN^KISS^ neurons increased, decreased, or showed no change (11.6 ± 2.9 events/cell/h pre‐drug, 10.2 ± 3.5 events/cell/h in bicuculline; *p* = .6875; Wilcoxon, *W* = −5). Similarly, the frequency of mSEs (1.9 ± 0.4 mSEs/cell/h pre‐drug, 2.5 ± 0.7 mSEs/cell/h in bicuculline; *p* = .6875; Wilcoxon, *W* = 5) and the numbers of cells contributing to each mSE did not change from 32.1% to 27.1% (4.1 ± 0.7 cells/mSE pre‐drug, 3.4 ± 0.4 cells/mSE in bicuculline; *p* = .4688; Wilcoxon, *W* = −8) (Figure 4B–D).

The same result was found in response to GABA_B_ receptor antagonism in five slices (5 mice) with CGP‐35348 (100 μM) having no significant effect on the numbers of calcium events (11.1 ± 2.9 events/cell/h pre‐drug, 8.0 ± 1.5 events/cell/h in CGP; *p* = .6250; Wilcoxon, *W* = −5), rate of mSEs (2.4 ± 0.7 pre‐drug, 1.9 ± 0.4 in CGP; *p* = .8125; Wilcoxon, *W* = −3), or numbers of ARN^KISS^ neurons contributing to each mSE (24.4%; 3.3 ± 0.7 cells/mSE pre‐drug versus 22.7%; 2.8 ± 0.2 cells/mSE in CGP; *p* = .6250; Wilcoxon, *W* = −5) (Figure 4E–H).

### 3.5 Effects of GABA_A_ and GABA_B_ receptor modulation on the dynamics of ARN^KISS^ neuron transients

In addition to modulating the frequency of burst events and mSEs, it is possible that GABA receptor transmission may modulate the dynamics of each burst of ARN^KISS^ neuron activity. This can be assessed by interrogating the shape of individual calcium events^3^ in experiments where transients were observed in the drug‐applied interval (5–6 slices/drug; Table 1). We found muscimol application led to an increase in both the half‐width (26.0 ± 4.0 s pre‐drug, 40.4 ± 7.0 s in muscimol; *p* = .0312; Wilcoxon, *W* = 21) and decay time (11.2 ± 1.4 s pre‐drug, 15.5 ± 1.6 s in muscimol; *p* = .0312; Wilcoxon, *W* = 21), but not rise time, of calcium events. No significant effects of GABA or other GABA_A_ or GABA_B_ receptor agonists or antagonists were detected on the dynamics of GCaMP transients exhibited by ARN^KISS^ neurons with the width, rise time, or decay time of transients (all *p* > .05, Wilcoxon) (Table 1).

## 4 DISCUSSION

We find here that GABA and GABA_A_ and GABA_B_ receptor activation result in an overall decrease in the frequency at which individual ARN^KISS^ neurons exhibit episodes of burst firing and that this impacts upon their ability to generate synchronized episodes of activity with other ARN^KISS^ neurons. Muscimol could also exert a biphasic effect wherein baseline calcium levels were initially increased before cells became less active. In stark contrast to glutamatergic signaling,^3^ there was no evidence that endogenous GABA transmission contributes to the individual burst firing or mSE behavior of ARN^KISS^ neurons in the brain slice.

The activation of GABA_A_ receptors resulted in a uniform suppression of burst firing and synchronicity amongst the population. However, in some cases, an immediate and transient increase in basal GCaMP fluorescence was detected upon entry of muscimol to the bath. This initial increase may reflect the observation that ARN^KISS^ neurons maintain a relatively high intracellular chloride concentration and that, in estradiol‐treated ovariectomized mice, this results in the outflow of chloride ions and membrane depolarization upon GABA_A_ receptor activation.^11^ As such, one possible scenario explaining the initial increase in GCaMP signal is that the small GABA_A_ receptor depolarization is greatly potentiated by on‐going glutamate‐AMPA receptor transmission to evoke intense firing and depolarization within the network. This overactive state could then lead to the subsequent inactivation of voltage‐gated sodium channels to inhibit action potentials.^15^ Another possibility for the baseline increase in calcium signal is that it may arise from potentially complex patterns of indirect input activation and inactivation within the brain slice. Ultimately, however, we note that the effect of GABA itself is only inhibitory, suppressing both the frequency of burst firing in individual neurons and their ability to synchronize. This indicates that the activation of GABAergic projections innervating ARN^KISS^ neurons would suppress their synchronized output. Notably, the sustained chemogenetic activation of agouti‐related protein/neuropeptide Y/GABA neurons that project directly to ARN^KISS^ neurons to release GABA results in suppressed LH secretion and disrupted estrous cyclicity.^9^, ^16^

The activation of GABA_B_ receptors requires sustained GABA release and results in relatively long‐lasting inhibition through G‐protein activation of postsynaptic potassium channels and the inhibition of pre‐ and postsynaptic calcium channels.^17^, ^18^ Given that most ARN^KISS^ neurons express transcripts for both of the GABA_B_ receptor subunits,^19^, ^20^ it is not unexpected to find that baclofen suppresses the frequency of their bursting behavior as observed through GCaMP transients. This may result from direct hyperpolarization at the level of the cell body and/or the suppression of on‐going glutamate release from their recurrent collateral innervation. Surprisingly, these effects on individual bursting activity did not significantly reduce synchronization levels between ARN^KISS^ neurons, although both the frequency of mSEs and the numbers of neurons contributing to each mSE were close to the 5% significance level. This might be due to insufficient activation of the GABA_B_ receptor by baclofen although we note that the 100 μM concentration used is well above the IC_50_ for baclofen and effective in suppressing GnRH neuron activity in brain slices.^21^, ^22^

We show that GABA_B_ receptor activation can suppress the activity of ARN^KISS^ neurons but note that the functional role of this type of GABAergic transmission is unknown. The embryonic deletion of GABA_B_ receptor signaling from all kisspeptin neurons has not been found to have any impact on fertility in male or female mice.^20^, ^23^ However, the suppression of GABA_B_ transmission globally or in all kisspeptin neurons has been reported to result in abnormal *Kiss1* and *Tac2* mRNA expression.^23^, ^24^, ^25^ Clearly, future studies will be required to address the role of GABA_B_ receptors expressed by ARN^KISS^ neurons in relation to the operation of the GnRH pulse generator.

An episode of burst firing by an individual ARN^KISS^ neuron in vivo generates an increment in GCaMP6s fluorescence that lasts from 30 to 130 s with a half maximum time of ~30 s.^3^, ^4^ This is comprised of a quickly rising peak with a half maximum time of ~9 s followed by a slower decay with a half maximum time of ~15 s.^3^ These in vivo parameters are the same as those reported here for individual ARN^KISS^ neuron GCaMP transients in the brain slice (Table 1). Interestingly, muscimol causes an increase in GCaMP transient half‐width and decay time whereas calcium burst dynamics exhibited by individual ARN^KISS^ neurons do not change when their frequency is reduced by GABA or baclofen. This suggests that the pattern of ARN^KISS^ neuron bursting may occur in a stereotypical all or nothing manner whereby, once initiated, the course of a burst remains relatively independent, except perhaps under conditions of GABA_A_ receptor membrane depolarization. The relative constancy of burst firing is also apparent when examining ARN^KISS^ neuron population SE dynamics in vivo. For example, the direct hyperpolarization of ARN^KISS^ neurons by noradrenaline reduces the frequency of population SEs but does not alter their duration or dynamics.^6^

These studies have been undertaken in slices obtained from diestrous female mice. Whether the same effects of GABA transmission on ARN^KISS^ neurons are present in males is unknown. Although the great majority of studies examining the GABA_A_ receptor to date have been undertaken in female mice, there is evidence that GABA_B_ receptors are expressed by ARN^KISS^ neurons in both sexes.^19^, ^20^ Sex differences have been found in the effects of fasting on GABA_A_ receptor postsynaptic potentials displayed by ARN^KISS^ neurons.^26^ It is also unknown whether the effects of GABA transmission on ARN^KISS^ neurons may vary across the estrous cycle. The expression of transcripts for multiple GABA_A_ and GABA_B_ receptor subunits is changed by treating OVX mice with estradiol,^19^ as are GABA_A_ receptor PSCs recorded from ARN^KISS^ neurons.^11^ Whether the more subtle changes in circulating estradiol that occur during the estrous cycle^27^ would impact on GABA_A_ and GABA_B_ receptor expression by ARN^KISS^ neurons remains to be investigated.

In summary, we provide evidence indicating that GABA release upon ARN^KISS^ neurons will inhibit their burst firing and ability to synchronize with other kisspeptin neurons through both GABA_A_ and/or GABA_B_ receptors. However, the brief activation of GABA inputs may facilitate their firing through GABA_A_ receptor‐mediated depolarization. Although the functional roles of GABAergic inputs to ARN^KISS^ neurons remain poorly understood, the present data suggest that they have considerable potential to modulate the frequency of pulse generator operation.

## AUTHOR CONTRIBUTIONS

**Toby Eddleston:** Methodology; investigation; analysis; writing – review and editing. **Paul G. Morris:** Methodology; investigation; software; analysis; writing – review and editing. **Allan E. Herbison:** Conceptualization; funding acquisition; writing – original draft, writing – review and editing; project administration; supervision.

## CONFLICT OF INTEREST STATEMENT

The authors declare no conflicts of interest.

## ETHICS STATEMENT

All experimental protocols were approved by the University of Cambridge Animal Welfare and Ethics Review Body under the UK Home Office license P174441DE.

## Data Availability

The data that support the findings of this study are openly available in Apollo‐University of Cambridge Repository at https://www.repository.cam.ac.uk/home.
